# The Immunoexpression of YAP1 and LATS1 Proteins in Clear Cell Renal Cell Carcinoma: Impact on Patients' Survival

**DOI:** 10.1155/2018/2653623

**Published:** 2018-04-03

**Authors:** J. Godlewski, J. Kiezun, B. E. Krazinski, Z. Kozielec, P. M. Wierzbicki, Z. Kmiec

**Affiliations:** ^1^Department of Human Histology and Embryology, School of Medicine, Collegium Medicum, University of Warmia and Mazury in Olsztyn, Olsztyn, Poland; ^2^Department of Pathomorphology, School of Medicine, Collegium Medicum, University of Warmia and Mazury in Olsztyn, Olsztyn, Poland; ^3^Department of Histology, Medical University of Gdansk, Gdansk, Poland

## Abstract

The aim of the study was to determine by immunohistochemistry cellular localization and immunoreactivity levels of YAP1 and LATS1 proteins in paired sections of tumor and unchanged renal tissues of 54 clear cell renal cell carcinoma (ccRCC) patients. Associations between clinical-pathological and overall survival (OS; median follow-up was 40.6 months) data of patients and YAP1 and LATS1 immunoreactivity were analyzed by uni- and multivariate Cox regression model and log-rank test. YAP1 immunoreactivity was found in the nuclei of tumor cells in 64.8% of ccRCC patients, whereas only 24.1% of tumors revealed cytoplasmic YAP1 expression. LATS1 immunoexpression was observed only in the cytoplasm of tumor cells in 59.3% of patients. LATS1 immunoreactivity in cancer cells negatively correlated with the size of primary tumor. The overall YAP1 immunoreactivity did not correlate with clinical-pathological data of patients. However, the subgroup of ccRCC patients who presented with cytoplasmic YAP1 immunoexpression had significantly shorter OS (median = 26.8 months) than patients without cytoplasmic YAP1 expression (median undefined). Multivariate Cox analysis revealed that increased cytoplasmic YAP1 (HR = 4.53) and decreased LATS1 immunoreactivity levels (HR = 0.90) were associated with worse prognosis, being independent prognostic factors. These results suggest that YAP1 and LATS1 can be considered as new prognostic factors in ccRCC.

## 1. Introduction

Renal cell carcinoma (RCC) is the most common type of cancer of the urinary tract. According to the latest worldwide registry data, 337,860 new cases (123,936 women and 213,924 men) with the total mortality of 143,406 cases (52,604 and 90,802 resp.) were reported in 2012 [1]. The RCC encompasses a group of heterogeneous tumors which originate from the renal tubular epithelial cells. The most frequent type of RCC is the clear cell renal cell carcinoma (ccRCC). It originates from the proximal tubular epithelium and is characterized by the worst clinical course and prognosis among other RCC types [2]. The genetic and epigenetic background of alterations that occur during development and progression of ccRCC has not been fully elucidated so far.

Yes-associated protein* 1* (YAP1) may be considered as one of the oncoproteins that play an important role in ccRCC pathogenesis, since deregulation of this gene (either at mRNA or protein level) was associated with progression of other malignancies [3–5].* YAP1* gene is located at 11q22 chromosome and this* locus* is amplified in many tumors [6].* YAP1*, as an oncogene, plays a role in cells' proliferation and furthermore it is also involved in other mechanisms of cancer progression such as epithelial-mesenchymal transition, cell migration, and formation of metastasis [7]. Moreover, numerous studies have shown higher* YAP1* gene expression as well as higher level of YAP1 protein in numerous cancers such as non-small-cell lung, breast, colorectal, and liver cancers [8].

YAP1 protein is a transcriptional coactivator which does not contain a DNA-binding domain; however, it interacts with transcription factors such as TEA domain (TEAD1–4) proteins binding to genes' promoters. Such a functional complex, composed of YAP1 and TEAD1–4 proteins, promotes expression of genes which are associated with cellular progression and proliferation (e.g.,* CTGF*,* Cyr61*,* Myc*, and* Gli*) [9, 10]. During physiological conditions, YAP1/TEAD complex acts as the transcriptional effector of the Hippo pathway that during development affects organs' size by inhibition of cell proliferation and activation of apoptosis [11]. The crucial component of the Hippo pathway is serine/threonine-protein kinase 1 (LATS1) that forms Hippo core protein cassette (together with MST2 protein) [12, 13]. Hippo pathway negatively regulates YAP1 translocation to nucleus via LATS1-dependent phosphorylation of YAP1 which in turn binds to cytoplasmic 14-3-3 protein. As a consequence the complex YAP1/14-3-3 is targeted for proteasomal degradation [14]. The translocation of YAP1 from the cytoplasm to the nucleus is necessary for its function as transcriptional coactivator. In this way the Hippo pathway components control and may inhibit cellular growth. In our previous study we found at both mRNA and protein levels that* LATS1* gene expression was downregulated and* YAP1* expression upregulated in ccRCC tumor tissues in comparison to corresponding samples of unaltered kidney tissues [13]. However, the cellular localization and expression of both proteins by immunohistochemistry (IHC) was not performed, and the studies of other authors provided opposite findings [15, 16]. Therefore, we decided to assess the immunoreactivity of LATS1 and YAP1 proteins within the cancer and normal kidney tissue of patients with ccRCC. The results of the IHC study were correlated with the clinical and pathological features of ccRCC patients. The postoperative follow-up was performed in order to evaluate the immunoexpression of the investigated proteins as possible risk factors of cancer progression and patients' survival.

## 2. Material and Methods

### 2.1. Clear Cell RCC Patients, Specimen Collection, and Ethics Statement

This study was carried out in accordance with the Declaration of Helsinki (1964). All procedures were approved by the Bioethics Committee for Scientific Research at the University of Warmia and Mazury in Olsztyn, (decision number 4/2010). Appropriate written informed consent regarding the use of tissue was obtained from each patient in the study. The fragment of postoperative tumor tissue and unchanged kidney tissue were obtained from 54 ccRCC patients (23 females and 31 males) with a mean age 64.07 ± 9.10, range 44–83 years, who underwent surgery at the Department of Oncological Surgery, Warmia and Mazury Oncological Center, Olsztyn, Poland, in the period between March 2010 and May 2014. None of the patients had suffered from a second neoplastic disease or other serious disease. The clinical characteristics and overall survival (OS) data of the patients were collected during the study and the median time of follow-up was 40.6 months. The tumor stage was characterized according to the TNM system (American Joint Committee on Cancer) [17]. Hematoxylin and eosin- (H&E-) stained sections of collected tumor and matching kidney specimens were evaluated by a pathologist to confirm their cancer or cancer-free phenotype, respectively. The degree of tumor malignancy was determined using the Fuhrman nuclear grading system [18]. The studied tissues were placed in 4% buffered formaldehyde, postfixed, dehydrated, embedded in paraffin, and cut into 5 *μ*m thick sections.

### 2.2. Immunohistochemistry

Immunohistochemical (IHC) analysis was performed as described previously by Godlewski et al. [20] with some modification. The sections were subjected to an antigen retrieval procedure by microwaving in Retrieval Solution Buffer (pH 6.0; Leica, Wetzlar, Germany), incubated with 3% H_2_O_2_ in methanol for 30 min, and then in commercial normal horse serum (Vector Laboratories, Inc., Burlingame, CA, USA) for 30 min. The sections were incubated overnight at 4°C with rabbit polyclonal anti-human antibodies against YAP1 (diluted 1 : 800 in PBS; #ab81183; Abcam, Cambridge, UK) or LATS1 (diluted 1 : 800 in PBS; #LS-C98952, LifeSpan Biosciences, Seattle, WA, USA) and then with secondary antibodies (commercially diluted; ImmPRESS Universal Reagent Anti-Mouse/Rabbit Ig; Vector Laboratories, Inc., Burlingame, CA, USA) for 30 min. To visualize the immunoreaction, the sections were immersed in 3,3′-diaminobenzidine (DAB; Dako, Glostrup, Denmark) and counterstained in Mayer's hematoxylin. The tissue sections were dehydrated in ethanol, cleared in xylene, and mounted. The specificity of the IHC staining was determined by omitting the primary antibody and replacing it with the same dilution of horse serum. The labelled tissues were photographed using a XC-50 camera (Olympus Corporation, Tokyo, Japan) mounted on a light microscope (BX-41; Olympus Corporation). Concomitantly to IHC, the H&E staining was performed to assess tissue morphology.

### 2.3. Evaluation of Immunohistochemical Reactions

IHC reactions for YAP1 and LATS1 in ccRCC tumors and corresponding normal kidney tissue were evaluated by pathologist who was blinded to the patients' clinical data. The scoring system for YAP1 nuclear immunoreactivity was based on the percentage of YAP1-immunoreactive cells: 0 for negative, 1 for up to 25%, 2 for up to 75%, and 3 for more than 75% of YAP1-immunoreactive cells. The intensity of YAP1 cytoplasmic IHC reactions was scored as follows: 0 for negative, 1 for weak, 2 for moderate, and 3 for strong. The scoring system for LATS1 cytoplasmic immunoreactivity was based on the percentage of LATS1-immunoreactive cells and the intensity of IHC reaction according to the immunoreactive score system (IRS) of Remmele and Stegner [19]. The scale is based on the percentage of cells showing positive reaction, 1 point: 1–10% cells, 2-points: 11–50%, 3 points: 51–80%, and 4 points: over 80% cells with positive reaction, as well as reaction intensity (0: no reaction, 1 low intensity reaction, 2: moderate intensity reaction, and 3: intense reaction). The final score depended on both parameters, percentage of positive cells and intensity of the reaction, and ranged from 0 to 12 points.

### 2.4. Statistical Analysis

Statistical analysis was carried out using Prism 6 (GraphPad, La Jolla, CA, USA) and Statistica 13 (StatSoft, Tulsa, OK, USA) software. Significance of differences between expression levels of analyzed proteins in ccRCC cells and proximal convoluted tubules (PCT) epithelial cells was tested by the Wilcoxon matched-pairs signed rank test. Correlations between the immunoexpression of the studied proteins and between levels of protein expression and clinical-pathological data were evaluated using Fisher exact test and Spearman's correlation test. Patients' OS fractions were calculated and visualized according to the Kaplan-Meier method and the statistical significance of differences in survival between groups of patients was evaluated by log-rank test. The univariate and multivariate associations of clinical-pathological data with patients' survival and immunoreactivity were assessed using Cox proportional hazards regression model. Differences were considered statistically significant for *p* < 0.05.

## 3. Results

### 3.1. Tumor ccRCC Cells Exhibit Altered YAP1 Immunoreactivity That Does Not Correlate with Clinical-Pathological Data of the Patients

**Figure 1:**
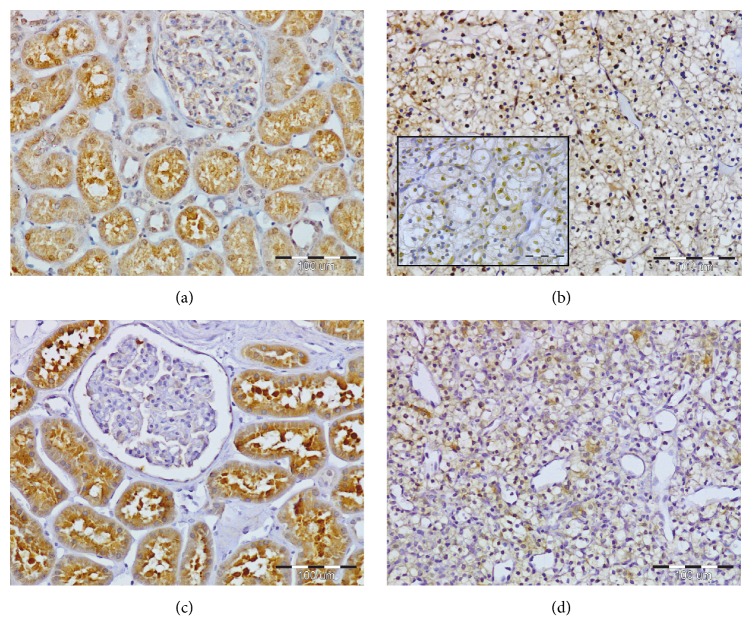
Evaluation of Yes-associated protein 1 (YAP1) and serine/threonine-protein kinase 1 (LATS1) expression levels in the sections of clear cell renal cell carcinoma (ccRCC) and unchanged kidney tissues by immunohistochemistry. In unchanged kidney cortex (a) strong YAP1 immunoreactivity was localized mainly in the cytoplasm of epithelial cells of proximal convoluted tubules (PCT). In ccRCC tumor cells YAP1 was expressed mainly in cell nuclei ((b) insert); however, weak to moderate YAP1 immunoreactivity was also observed in the cytoplasm of tumor cells in 13/54 ccRCC patients (b). Strong LATS1 immunoreactivity was present in the cytoplasm of PCT epithelium (c) and weak immunoreactivity in the cytoplasm of ccRCC cells (d), respectively. Magnifications: (a)–(d) ×200; insert (b) ×300.

**Figure 2:**
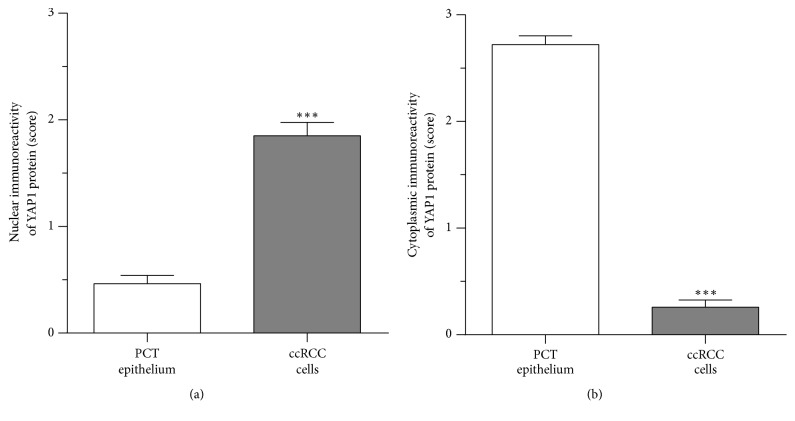
Comparison of Yes-associated protein 1 (YAP1) expression in the clear cell renal cell carcinoma (ccRCC) and normal kidney tissues assessed by immunohistochemistry. The levels of nuclear (a) and cytoplasmic (b) YAP1 immunoreactivity in the tumor cells are shown in relation to the levels of YAP1 immunoreactivity in the epithelial cells of proximal convoluted tubules (PCT) of normal kidney. Data are presented as the mean ± SEM (*n* = 54). *p* values were calculated using the Wilcoxon matched-pair test. ^*∗∗∗*^*p* < 0.001 (see Methods).

YAP1 protein immunoreactivity was found in both ccRCC tumor and normal kidney sections. However, ccRCC cells exhibited predominantly nuclear YAP1 immunoreactivity (Figures 1(a) and 1(b), insert), whereas epithelial cells of the PCT were characterized by predominant cytoplasmic expression of YAP1 (Figure 1(a)). The nuclear immunoreactivity of YAP1 was moderate to strong in 35/54 (64.8%) and absent or weak in 19/54 (35.2%) of ccRCC specimens. In 75.6% of ccRCC patients YAP1 immunoreactivity was absent in the cytoplasm of tumor cells; however, in 13/54 (24.1%) of ccRCC patients tumor cells exhibited weak to moderate cytoplasmic YAP1 immunoreactivity (Figure 1(b)). In comparison to the respective cellular compartments of PCT cells of normal kidney the immunoexpression of YAP1 was significantly increased in the nuclei (Figure 2(a)) and decreased in the cytoplasm (Figure 2(b)) of ccRCC tumor cells.

YAP1 nuclear and cytoplasmic expression levels in the tumor cells did not correlate with demographic and clinical-pathological data of patients with ccRCC (Table 1).

### 3.2. LATS1 Immunoreactivity Is Decreased in ccRCC Cells and Is Associated with a Size of the Primary Tumor

**Figure 3:**
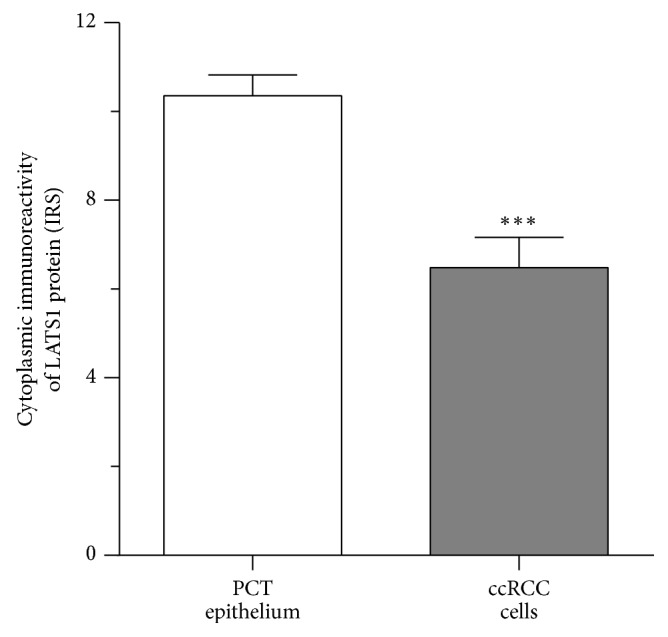
Comparison of serine/threonine-protein kinase 1 (LATS1) immunoreactivity in the clear cell renal cell carcinoma (ccRCC) and unchanged kidney tissue. The level of cytoplasmic LATS1 immunoreactivity in tumor cells is shown in relation to its immunoreactivity in the epithelial cells of proximal convoluted tubules (PCT) of unchanged kidney. Data are presented as the mean ± SEM (*N* = 54). *p* values were calculated using the Wilcoxon matched-pair test. ^*∗∗∗*^*p* < 0.001, as described in Methods.

LATS1 immunoexpression was found only in the cytoplasm of tumor cells and PCT epithelium (Figures 1(c) and 1(d)). LATS1 immunoreactivity was significantly lower in ccRCC cells as compared to the PCT cells of normal kidney (Figure 3). However, the intensity of LATS1 immunoreactivity varied between cancer cells since it was absent or weak in 22/54 (40.7%) and moderate to strong in 32/54 (59.3%) of ccRCC patients.

LATS1 immunoexpression in cancer cells negatively correlated with the size of primary tumor (Table 2).

### 3.3. YAP1 Nuclear Immunoreactivity Is Associated with YAP1 and LATS1 Presence in the Cytoplasm of ccRCC Cells

We found negative correlation between YAP1 nuclear and cytoplasmic immunoreactivity levels in cancer cells (*r* = −0.32, *p* = 0.0166). Moreover, cytoplasmic LATS1 immunoreactivity was positively associated with the nuclear localization of YAP1 immunoexpression (*r* = 0.34, *p* = 0.0127).

### 3.4. Overall Survival of ccRCC Patients Is Associated with the Immunoreactivity of YAP1 and LATS1 Proteins in ccRCC Tumor Cells

**Figure 4:**
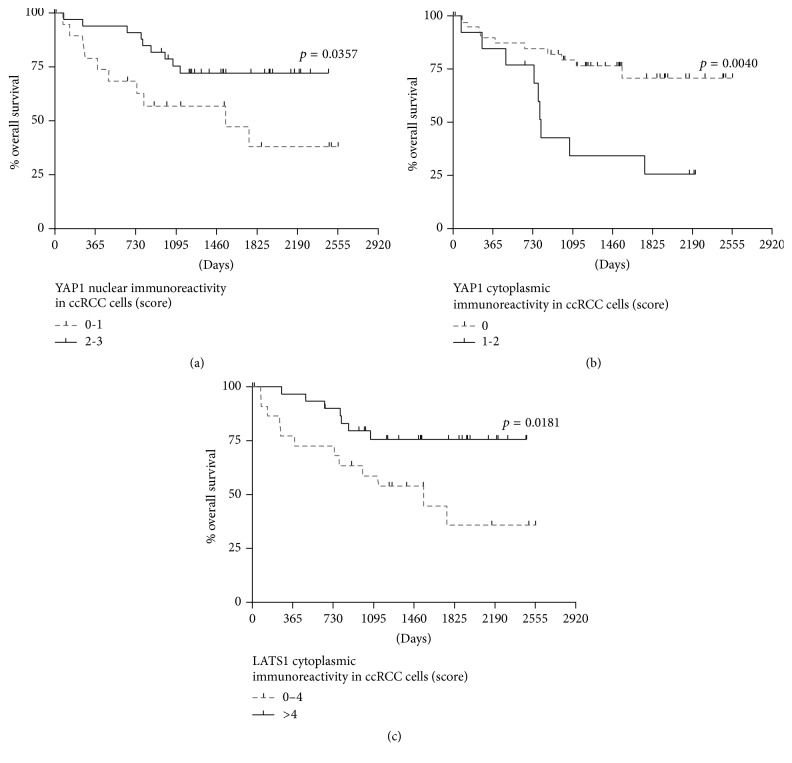
Analysis of overall survival of the patients. Kaplan-Meier curves of 54 ccRCC patients regarding the Yes-associated protein 1 (YAP1) nuclear (a), YAP1 cytoplasmic, (b) and serine/threonine-protein kinase 1 (LATS1) cytoplasmic (c) immunoreactivity levels. The scores of immunoreactivity were determined as described in Methods; *p* values were calculated using the log-rank test.

Kaplan-Meier plots presenting the ccRCC patients survival and immunoreactivity of investigated protein are demonstrated in Figure 4. Lower nuclear YAP1 presence was associated with shorter survival of ccRCC patients (median = 50.0 months versus undefined for high nuclear YAP1 expression, Figure 4(a)). On the contrary, the subgroup of 25% of ccRCC patients in whom cytoplasmic presence of YAP1 in cancer cells was observed showed significantly shorter OS (median = 26.8 months) than patients without cytoplasmic YAP1 presence (median undefined) (Figure 4(b)). Also decreased cytoplasmic LATS1 immunoreactivity was associated with significantly shorter survival (Figure 4(c)). Univariate Cox proportional hazards regression revealed that increased YAP1 and decreased LATS1 immunoreactivity levels in the cytoplasm of ccRCC cells were significantly associated with worse prognosis (Table 3). Multivariate regression analysis confirmed that the expression levels of YAP1 and LATS1 proteins (HR = 4.53 and HR = 0.90, resp.), along with the presence of distant metastases (HR = 4.94), are independent prognostic factors in ccRCC (Table 3).

## 4. Discussion

YAP1 is an activator of TEAD1–4 transcription factors and it has been found that TEAD1–4 family members play important role in tumor progression via activating progression-inducing genes (CTGF, Cyr61, and Myc) [9, 10]. The role of YAP1 in the stimulation of cell proliferation is crucial during organogenesis and this function may be inhibited by the Hippo pathway components by cytoplasmic sequestration of YAP1 after its phosphorylation by LATS1 kinase [11, 12]. The IHC studies published so far underline the role of YAP1 in the course of several types of cancers of the alimentary tract and demonstrate the correlation between higher expression of YAP1 protein, cancer development, and poor patients' prognosis [21–23].

Our IHC study revealed predominant YAP1 protein localization within nuclei of ccRCC cells in contrast to the predominantly cytoplasmic localization in the cells of normal kidney tissue. Moreover, nuclear YAP1 immunoreactivity was significantly higher in ccRCC cells than in the nuclei of proximal tubules of histologically unaltered kidney cortex. These observations are in concordance with our previously performed analysis of the* YAP1* expression at the mRNA and protein levels by QPCR and Western blotting techniques, respectively. In that study we showed that* YAP1* gene upregulation and worse clinical outcome of ccRCC patients as well as higher YAP1 protein expression in cancer tissue homogenates were associated with the clinical stage and pathomorphological features such as higher TNM and Fuhrman's stages [13].

In ccRCC the immunoreactivity of YAP1 has been studied by only few authors. Cao et al. found that 63.3% (19/30) of ccRCC patients exhibited positive YAP1 immunoexpression, whereas the positive rate of YAP protein expression in the normal tissues was 33.3% (10/30) [15]. In contrast to our findings, they observed correlation between the protein presence in cancer tissues, low differentiated type of cancer, and advance stage of the illness [15]. However, these authors did not differentiate patients in relation to the intensity of YAP1's nuclear or cytoplasmic immunoreactivity as presented in our study. On the contrary, Hu et al. reported lower YAP protein immunoreactivity in tissue microarrays of 75 ccRCC patients that was correlated with more advanced T feature and more advanced clinical stage of cancer [16]. However, in the latter study no data about patients survival were provided and the analysis of the microphotographs presented in the latter study is difficult since the applied magnifications (×10) do not allow for the exact assessment.

The meta-analyses of 20 IHC studies related to YAP1 presence in cancer cells showed that nuclear as well as overall (nuclear and cytoplasmic) localization of this protein correlated with poorer overall survival (OS) time and disease-free survival time (DFS) in numerous types of cancer [24]. In the present study we demonstrated predominant nuclear YAP1 localization in ccRCC cells which may correspond to the YAP1's role as an oncoprotein which stimulates cell proliferation. Interestingly, the subgroup of ccRCC patients in whom cytoplasmic presence of YAP1 in cancer cells was observed exhibited the strongest correlation with patients' poor prognosis. The Cox statistical test, independently of Kaplan-Meier analysis, revealed high hazard ratio of death of ccRCC patients demonstrating cytoplasmic YAP1 presence. This is the first prognostic observation of this kind that is of a potential importance for the assessment of the clinical status of the ccRCC patients. Our finding is supported by the results of several studies, which suggest that in the cytoplasm of cancer cells YAP1 may play a role as a molecular factor inducing more aggressive cancer behavior [25]. It has been shown that both YAP1 and KRAS have a common biding site which is the E2F transcription factor and that in the absence of KRAS signaling YAP1 functionally replaces KRAS in KRAS-dependent cancer cells [26]. It also is possible that not all of the cytoplasmic YAP1's molecules become degraded and some of them, or phosphorylated YAP1 protein, interact with the cell signaling pathways other than Hippo pathway that may stimulate proliferation of cancer cells. The oncogenic role of YAP1 may depend on the biology of primary cancer type. For instance, in liver tumors (hepatocellular carcinoma and cholangiocarcinoma) YAP1 cytoplasmic expression was associated with keratin 19 expression which was associated with cancer aggressiveness and patients' poor prognosis [27]. In squamous cell carcinoma of the uterine cervix the level of YAP cytoplasmic immunoreactivity was shown to be associated with histological grade, formation of lymph node metastasis, and cancer recurrence [28].

In our IHC study the presence of LATS1 was observed only within the cytoplasm of normal and cancer cells, although in 40% of patients no or weak immunoreactivity was found in ccRCC cells. Such localization corresponds to the well-known enzymatic function of LATS1 which phosphorylates and, thus, inactivates YAP1 protein in the cytoplasm [14]. In an immunohistochemical study lower LATS1 protein expression was found in renal cancer tissue and it correlated with the clinical stage and pathological grade of ccRCC [29]. Decreased level of LATS1 immunoreactivity in ccRCC cells, as observed in the present study, corresponds to the analysis of* LATS1* gene expression on another set of ccRCC patients where we observed strong association between decreased LATS1 mRNA and protein contents in cancer tissue lysates [13]. We also identified the possible epigenetic mechanism of decreased LATS1 level: the hypermethylation of LATS1 promoter region was strongly associated with lower LATS1 content [13]. Although we did not check the association between LATS1 methylation and YAP1 protein content in our previous study, other authors in the study of ccRCC 786-O cell line observed that the demethylation of LATS1 promoter was strongly associated with upregulation of YAP1 protein in cells [29]. Since the relationship between epigenetic regulation of LATS1 (DNA methylation-mRNA-protein) and its influence on YAP1 protein cellular presence was verified, we suppose that IHC is a reliable method to assess LATS1 and YAP1 expression in ccRCC. Also the TCGA data obtained for 469 ccRCC tumors shows altered* YAP1* mRNA and* LATS1* mRNA level in cancer tumors.* YAP1* mRNA was upregulated in 9.6% and downregulated in 4.9% cases and* LATS1* mRNA was upregulated in 4.5% and downregulated in 10.7% cases, when a-*z* score threshold was set for 1.5-fold change [30].

The present study lacks the statistical association of molecular data with the PFS observations due to insufficient outcome data of patients. However, there are only two studies that analyzed the influence of YAP1 expression on PFS in ovarian cancer (OC) [31] and colorectal cancer (CRC) [32]. It was shown that single-nucleotide polymorphism (SNP) which affects the upregulation of YAP1 gene was associated with OS and PFS in the study on 2901 OC as well as data of 4434 cases in TCGA [31]. The microarray study on 1028 patients with CRC indicated that high YAP1 mRNA levels were associated with shorter PFS and the upregulation of YAP1 mRNA was an independent prognostic factor of CRC (HR = 1.82, *p* = 0.034) [32]. Moreover, in the group of 36 mantle-cell lymphoma patients decreased LATS1 expression correlated with PFS and OS [33]. Such results cannot be compared with our data, since the expression of YAP1 and LATS genes was assessed at different levels: DNA [31] and mRNA [32, 33], whereas we analyzed the immunolocalization of protein products in the present study.

## 5. Conclusion

The obtained results related to YAP1 and LATS1 immunoexpression in the cytoplasm of ccRCC cells suggest that immunohistochemical assessment of YAP1 and LATS1 status could be used as a prognostic marker and might be helpful in identifying high-risk ccRCC patients.

## Figures and Tables

**(a):** 

Qualitative parameters	Number of patients	YAP1 nuclear immunoreactivity in ccRCC			YAP1 cytoplasmic immunoreactivity in		
		cells (expressed as scores)^*∗*^			ccRCC cells (expressed as scores)^*∗∗*^		
		0-1 *n* (%)	2-3 *n* (%)	*p* value	0 *n* (%)	1-2 *n* (%)	*p* value
Total	54	19 (35.2)	35 (64.8)		41 (75.9)	13 (24.1)	
Men	31	11 (35.5)	20 (64.5)	1.0000^a^	24 (77.4)	7 (22.6)	1.0000^a^
Women	23	8 (34.8)	15 (65.2)		17 (73.9)	6 (26.1)	
Depth of invasion							
T1 + T2	42	13 (31.0)	29 (69.0)	0.3066^a^	30 (71.4)	12 (28.6)	0.2538^a^
T3	12	6 (50.0)	6 (50.0)		11 (91.7)	1 (8.3)	
Fuhrman grade							
G1 + G2	42	12 (28.6)	30 (71.4)	0.0867^a^	34 (81.0)	8 (19.0)	0.1340^a^
G3	12	7 (58.3)	5 (41.7)		7 (58.3)	5 (41.7)	
Distant metastases							
M0	47	16 (34.0)	31 (66.0)	0.6866^a^	35 (74.5)	12 (25.5)	1.0000^a^
M1	7	3 (42.9)	4 (57.1)		6 (85.7)	1 (14.3)	

**(b):** 

Quantitative parameters	*r*		*p* value		*r*		*p* value
Age	−0.08		0.5494^b^		−0.01		0.9256^b^
Tumor size	−0.08		0.5544^b^		0.06		0.6581^b^

^*∗*^The scores evaluate percentage cells with YAP1 nuclear immunoreactivity. ^*∗∗*^The scores assess intensity of YAP1 cytoplasmic IHC reactions, both described in Methods. ^a^Fisher exact test. ^b^Spearman's correlation.

**(a):** 

Qualitative parameters	Number of patients	LATS1 immunoreactivity in ccRCC cells (expressed as IRS score)^*∗*^		
		0–4 *n* (%)	>4 *n* (%)	*p* value^a^
Total	54	22 (40.7)	32 (59.3)	
Men	31	13 (41.9)	18 (58.1)	1.0000^a^
Women	23	9 (39.1)	14 (60.9)	
Depth of invasion				
T1 + T2	42	15 (35.7)	27 (64.3)	0.1942^a^
T3	12	7 (58.3)	5 (41.7)	
Fuhrman grade				
G1 + G2	42	14 (33.3)	28 (66.7)	0.0507^a^
G3	12	8 (66.7)	4 (33.3)	
Distant metastases				
M0	47	18 (38.3)	29 (61.7)	0.4260^a^
M1	7	4 (57.1)	3 (42.9)	

**(b):** 

Quantitative parameters		*r*		*p* value
Age		0.09		0.5370^b^
Tumor size		−**0.39**		**0.0035** ^b^

^*∗*^IRS: semiquantitative evaluation of immunoreactivity (percentage of cells × intensity score) was performed according to Remmele and Stegner [19] as described in Methods. ^a^Fisher exact test. ^b^Spearman's correlation. RQ: relative quantification. Significant *p* values (<0.05) are given in bold.

**Table 3:** Uni- and multivariate analysis (Cox proportional hazards regression) of overall survival of patients with clear cell renal cell carcinoma.

Parameter	Univariate Cox regression		Multivariate Cox regression	
	HR (95% CI)	*p* value	HR (95% CI)	*p* value
YAP1 nuclear immunoreactivity	0.63 (0.38–1.02)	0.0625		
YAP1 cytoplasmic immunoreactivity	3.24 (1.49–7.03)	**0.0029**	4.53 (1.73–11.9)	**0.0022**
LATS1 cytoplasmic immunoreactivity	0.90 (0.81–0.99)	**0.0255**	0.90 (0.91–0.99)	**0.0289**

Gender (women versus men)	0.97 (0.39–2.42)	0.9525		
Age (years)	1.02 (0.97–1.07)	0.3958		
Tumor size (cm)	1.17 (1.03–1.34)	**0.0168**	1.12 (0.93–1.36)	0.2231
Depth of invasion (T3 versus T1 + T2)	2.03 (0.77–5.37)	**0.1526**		
Fuhrman grade (G3 versus G1 + G2)	4.07 (1.60–10.4)	**0.0033**	1.49 (0.56–3.97)	0.4299
Distant metastases (M1 versus M0)	4.35 (1.64–11.5)	**0.0032**	4.94 (1.59–15.3)	**0.0056**

Median follow-up time: 40.6 months; HR: hazard ratio; CI: confidence interval; RQ: relative quantification. Significant *p* values (<0.05) are given in bold.
